# Improving Emergency Department Staff Satisfaction Through Lean Thinking: Evidence From a Mixed Study

**DOI:** 10.1155/jonm/2158539

**Published:** 2026-03-20

**Authors:** Andrea Pastor-Puigdomènech, Miquel Sánchez, Cristina Font-Cabrera, Núria Fabrellas, Llúcia Benito-Aracil, Eva Maria Guix-Comellas

**Affiliations:** ^1^ Emergency Department, Clínic University Hospital, Barcelona, Catalonia, Spain; ^2^ Department of Fundamental and Clinical Nursing, Faculty of Nursing, University of Barcelona, Barcelona, Catalonia, Spain, ub.edu; ^3^ Emergency Care Research Group: Processes and Pathologies, August Pi i Sunyer Biomedical Research Institute, IDIBAPS, Barcelona, Catalonia, Spain, idibaps.org; ^4^ Emergency Department, Bellvitge University Hospital, Barcelona, Catalonia, Spain, bellvitgehospital.cat; ^5^ Bellvitge Institute of Biomedical Research, IDIBELL Nursing Research Group, Barcelona, Catalonia, Spain; ^6^ Department of Public Health, Faculty of Nursing, University of Barcelona, Barcelona, Catalonia, Spain, ub.edu

**Keywords:** emergency department, healthcare management, job satisfaction, Lean Thinking, mixed methods, quality improvement

## Abstract

**Aims:**

To compare job satisfaction among emergency department staff before and after the implementation of Lean Thinking and to explore staff experiences regarding this methodology, identifying associated satisfaction factors.

**Background:**

Lean Thinking, originally developed in the automotive industry, is increasingly applied in healthcare to optimise processes and improve patient care. Staff engagement and perceptions are key determinants of successful implementation.

**Methods:**

A mixed‐methods pre–post design was conducted. The Font‐Roja job satisfaction questionnaire was administered preintervention and 1 year postimplementation, and semistructured interviews were conducted with emergency department staff.

**Results:**

Overall job satisfaction increased (3.31–3.43; *p* = 0.029), with significant improvements in job monotony (3.30–3.64; *p* = 0.038) and physical work setting (3.31–3.75; *p* = 0.007). Qualitative analysis revealed ten subthemes including improved organisation and teamwork, a more methodical workflow, the need for adaptation, and concerns about potential dehumanisation.

**Conclusion:**

Lean Thinking increased job satisfaction in the emergency department, favouring structured work and better interprofessional communication. The professionals supported its continuity and highlighted the need for follow‐up meetings and constant improvements.

**Summary**
• Implications for the profession and patient care:
◦ Healthcare professionals are a key factor in implementing Lean Thinking. Continuous improvement of the service should be carried out through regular meetings and in conjunction with management to provide better care for patients.
• Impact:
◦ What problem did the study address?
◦ Job satisfaction and the experience of professionals in implementing Lean Thinking.
◦ What were the main findings?
◦ The results revealed the experiences of the hospital’s emergency department professionals in implementing Lean Thinking and identified areas for improvement. Healthcare professionals expressed a preference for working with lean methodology and adapting it to their work context. Work environment, quality care, leadership and training were also revealed as key factors in job satisfaction.
◦ Where and on whom will the research have an impact?
◦ The research will have an impact on future implementations of lean methodology, the functioning of hospital emergency departments and their staff.
• Reporting Method: The nonrandomized evaluation studies (TREND) and the Consolidated Criteria for Reporting Qualitative Research (COREQ) guidelines were followed.
• Patient or Public Contribution: No patient or public contribution.
◦ What does this paper contribute to the wider global clinical community?
◦ Provides information on the impact of lean thinking on the job satisfaction of healthcare professionals. Highlights the critical role of employee experiences in ensuring the successful implementation and sustainability of Lean Thinking in the emergency department.
◦ Identifies factors that influence the job satisfaction of healthcare professionals.
◦ Addresses an important gap in the literature by offering a mixed‐methods approach to comprehensively understand the impact of Lean Thinking for the healthcare workforce.

## 1. Introduction

Overcrowding in emergency departments (ED) is a significant problem affecting patients and healthcare professionals around the world [1]. In recent years, it has become evident that long waiting times are a major barrier to patient care, affecting patient safety and increasing mortality and healthcare costs [2]. In ED, saturation and workload often exceed resource availability. This leads to long waiting times, delays in acute care and prolonged hospital stays, and staff stress [3]. ED collapse results from an imbalance between the demand for emergency care and its response capacity [4], and this challenge is expected to grow as demand continues to rise [5].

Healthcare organisations face the challenge of identifying management solutions that improve efficiency, quality of care and staff satisfaction [6]. As a result, many healthcare managers consider it necessary to apply quality improvement models [7]. Implementing changes across the care process, integrating healthcare professionals at all levels, and optimising resource utilisation are essential to address ED overcrowding effectively [8].

## 2. Background

Lean is a management philosophy that originated in the Toyota system for car production lines. Widely used in the automotive industry, it has been adapted to the healthcare sector since 2002 with the aim of improving the quality of goods and services, reducing costs, increasing productivity and optimising resources by reviewing processes to create value for the patient [9]. Lean principles are a method of continuous process improvements that aim to create customer value, not only in what the customer wants but also in the speed with which it is delivered [10–12].

Several authors have investigated the process of implementing Lean Thinking (LT) in different healthcare settings: ED, pharmacy services, oncology, radiotherapy, primary care, operating rooms and mental health centres [13, 14]. Different approaches to its implementation have been identified, of which two stand out: the first with a more global vision, where LT is implemented as an integral organisational philosophy that systematically tackles waste, understood as ‘activities that do not add value’, at all levels [15]; the second, where LT is not implemented in a generic way, but as a toolbox, where specific practices are applied in small and concrete processes or units. [16]. Although the implementation of LT so far appears to be effective in the short term, there is little evidence of the sustainability of these benefits in the long term [17]. These approaches often overlook crucial aspects such as staff engagement, participation or satisfaction. While operational outcomes of Lean in healthcare are increasingly documented, its impact on staff satisfaction and experiences remains underexplored. Staff satisfaction is a critical factor in the success and sustainability of process improvement initiatives and can be conceptualised through theoretical frameworks such as Herzberg’s Two‐Factor Theory and the Job Demands‐Resources (JD‐R) model, which emphasise the importance of meaningful work, autonomy and supportive environments [18].

Few studies have used mixed‐methods approaches to capture both quantitative outcomes and qualitative staff perspectives, limiting understanding of how lean interventions influence employee satisfaction. For this reason, a mixed‐methods design enables linking measurable organisational effects with staff perceptions and experiences, providing a comprehensive view of the human dimension of lean and reinforcing the study’s contribution in addressing satisfaction, engagement and resistance [19]. A key factor in the successful implementation of LT in healthcare organisations is to look beyond the technical aspects. [20, 21]. Therefore, there is a clear gap in the literature regarding the effects of lean on staff satisfaction in emergency settings, particularly when examined through a rigorous mixed‐methods framework.

## 3. Aims

This study had two objectives: (a) to compare the level of job satisfaction of professionals working in the ED before and after the implementation of LT and (b) to explore the experiences and perceptions of nursing and medical staff in relation to the implementation of LT.

## 4. Methods

### 4.1. Design

#### 4.1.1. Mixed Methodology Study

**FIGURE 1:**
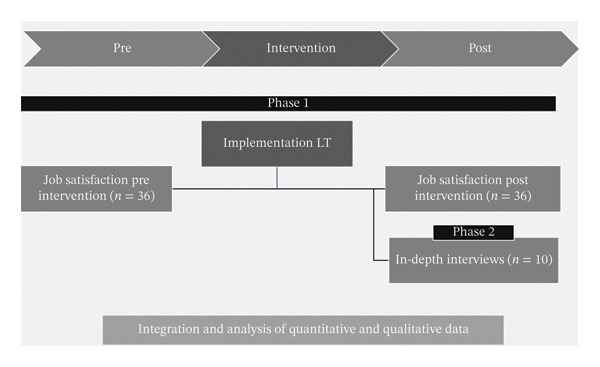
Schematic of the study design. Phase 1: quantitative; Phase 2: qualitative. LT = Lean Thinking.

A quasiexperimental, pre‐ and posttest design without a control group was carried out. The results of both phases were compared. Subsequently, a qualitative phenomenological study was conducted using semistructured in‐depth interviews (Figure 1). A mixed methods design provides a better understanding of the perspectives of key stakeholders. Mixed methods research provides a more comprehensive and enriching view, as it combines the strengths of both quantitative and qualitative approaches. It is recognised because it allows for a more in‐depth exploration of the phenomenon under study [22].

### 4.2. Setting

The study was conducted in the ED of the Hospital Clínic de Barcelona from March 2022 to May 2024. The hospital is a high‐tech hospital serving a population of 540,000 in the Barcelona region of Spain. It has a main site, which is a high‐tech hospital, and two smaller sites, which are low‐complexity centres, one of which is the Plató site where this study was conducted. This care centre only treats general emergencies in adult patients.

### 4.3. Subjects and Sample

#### 4.3.1. Subjects of the Quantitative Part

All ED workers who met the following selection criteria were included. Inclusion criteria were: nursing staff, physician staff, auxiliary healthcare staff, radiology technician staff and administrative staff; and signing the informed consent form. Exclusion criteria were having less than 6 months of work experience in the ED. Withdrawal criteria were that they left the study or ceased to work in the ED.

The total number of staff working in the ED was 51. Accepting an alpha risk of 0.05 and a beta risk of less than 0.2 for bilateral contrast, 24 subjects were needed, assuming a baseline event rate of 0.54 and a final event rate of 0.99. A loss‐to‐follow‐up rate of 20% was estimated. The type of sampling was nonrandomised by convenience [23]. The final sample obtained was 36 participants, which may have reduced statistical power.

#### 4.3.2. Subjects of the Qualitative Part

For the qualitative phase, each participant was selected for their potential to provide in‐depth and detailed information on the topic of interest. Key informants were selected, who voluntarily participated in the study. Sampling was purposive. A total of ten health professionals were interviewed. The profile of the selected participants was that they had at least 1 year of seniority, were nursing or physician staff, and had participated in the first quantitative phase of the study. Data saturation was used as the criterion for sample sufficiency and was reached at interview ten, at which point no new information or themes emerged.

### 4.4. Study Intervention

The implementation of the LT started in the last week of April 2022. In order to carry it out, the staff of the Hospital Clínic de Barcelona who were in possession of the LT certification were first contacted. These experts went to the workplace, i.e., the ED (called GEMBA after LT), to observe the daily operation of the ED. At the same time, all emergency staff were trained in the basic principles and tools of LT through 3 h workshops.

Then, during the following 2 months, a Value Stream Map (VSM) poster was physically displayed in the ED, containing a standard prototype of the future care process designed by the experts, according to the severity of the patient, with the intention of identifying activities that do not add value to care (called waste in LT). This VSM should also reflect barriers and solutions to create a new standard of care adapted to ED. The VSM is a tool to identify opportunities to reduce waste and integrate the steps of the process [24]. The VSM graphically represents the people, resources, activities and information flows required to provide appropriate healthcare. The VSM requires the cooperation of all staff involved to outline the sequence of the workflow and the time required for each procedure [25]. A simplified visual representation of the VSM used during implementation is included as Appendix 1. Once the VSM had been completed with the participation of all ED healthcare staff, a multidisciplinary team consisting of managers, physicians, nurses and administrative and healthcare support staff met to analyse all the information recorded in the VSM. A consensus was reached on a basic document that standardised the care process in the ED. These meetings were held regularly for monitoring and continuous improvement.

### 4.5. Instrument With Validity and Reliability/Data Source

The questionnaire used for the quantitative data collection consisted of a previously validated scale. The original Font‐Roja questionnaire is a version derived from the tool used in the Tecumseh Community Health Study [26, 27] in 1988. Although the Font‐Roja questionnaire is not specific to LT interventions, it is a validated and reliable instrument for measurement of job satisfaction in healthcare professionals in Spain and therefore represents the most appropriate tool for this context.

The original Font‐Roja questionnaire contained 24 questions grouped into nine dimensions of job satisfaction [28]. In 2006, the questionnaire was revised, and two new questions were added and grouped under a new dimension [29]. This extended questionnaire can explain 61.81% of the variance of job satisfaction and has an internal consistency of 0.791.

The 2006 Font‐Roja questionnaire, validated for use in Spain, consists of 26 items exploring 10 dimensions: job satisfaction (4 items), work‐related stress (5 items), professional competence (3 items), job pressure (2 items), professional promotion (3 items), interpersonal relationship with their superiors (2 items), interpersonal relationship with co‐workers (1 item), extrinsic characteristics of status (2 items), job monotony (2 items) and physical work setting (2 items) [30] (Table 1). Each question is rated on a Likert scale: strongly disagree (1), disagree (2), neither agree nor disagree (3), agree (4) and strongly agree (5) [23, 31].

**TABLE 1:** Dimensions explored by the Font‐Roja questionnaire [23].

Dimensions	Definition
1. Job satisfaction	Level of job satisfaction.
2. Work‐related stress	The level of stress induced by the worker’s job, as manifested by fatigue and perceived level of responsibility.
3. Professional competence	Degree of correspondence between vocational training and job position.
4. Job pressure	Degree of work overload in the workplace.
5. Professional promotion	Degree of aptitude for career advancement.
6. Interpersonal relationship with their superiors	Degree of knowledge of manager’s expectations of the employee.
7. Interpersonal relationship with co‐workers	Level of satisfaction from relationships with co‐workers
8. Extrinsic characteristics of status	Degree of recognition of work in terms of salary, trust, and independence
9. Job monotony	The degree of autonomy in relationships with coworkers and the lack of variety in the work performed.
10. Physical work setting	Degree of satisfaction with the physical and ergonomic characteristics of the work environment.

The overall job satisfaction is calculated by: Adding the score of the 26 items (the range is between 26 and 130) and dividing it by the items [31]. The score theoretically varies between 1 and 5 points, with 3 being the cut‐off point. Thus, employees with a score of 3 or above are considered ‘satisfied’, while those with a score below 3 are considered ‘dissatisfied’ [23]. There are items where higher scores indicate greater satisfaction (1, 2, 3, 4, 6, 13, 21, 23, 24, 25, 26), and others where the higher the scores, the lower the satisfaction (5, 7, 8, 9, 10, 11, 12, 14, 14, 15, 16, 17, 18, 19, 20, 22); for this reason, it is necessary to recode by adding the scale inversely (*y* = 6 − *x*) [27].

Sociodemographic variables and their employment status were also collected using an ad hoc questionnaire designed to collect the following: gender, age, length of professional experience, years in the ED, job position, work shift (morning, afternoon, evening, night, weekend and rotating) and type of employment contract (permanent and nonpermanent).

### 4.6. Data Collection

#### 4.6.1. Quantitative Data Collection

The questionnaire was administered before the intervention (April 2022) and 1 year after the implementation of the LT (May‐June 2023). After the workers signed the informed consent, they were given questionnaires (Font‐Roja and ad hoc) in a sealed envelope to always ensure confidentiality and anonymity. Once completed, the workers themselves returned the envelope to the principal investigator (PI).

#### 4.6.2. Qualitative Data Collection

Within the qualitative part of the research, a semistructured interview was used for data collection. The interviews were conducted face‐to‐face in a reserved room in the hospital by A.P‐P as a nurse researcher. The interviewer acknowledged the potential for insider bias and actively mitigated its influence by maintaining a neutral stance, engaging in reflexive discussions with the research team, and applying systematic coding procedures. The interviews lasted approximately 50 min. They were recorded on tape and then transcribed for proper analysis. The interview questions were grouped into four main categories: lean methodology, change in working practices, job satisfaction and organisational factors. The questions were formulated based on a literature review and the quantitative part of the study. Both interviews and transcripts were stored on the PI’s professional mail drive.

A total of 10 health professionals were interviewed: 6 nurses and 4 physicians. Privacy was always maintained so that participants felt free to share their feelings, ideas and beliefs without fear of reprisal. At the same time, the interviewees signed a research participation agreement (interview) document, giving their written consent to participate. After the interviews, the PI transcribed the audio content and notes taken during the interviews. Data collection ended after the tenth interview when key data became repetitive across participants, and no new themes of research interest emerged. The process continued until data saturation was reached [22, 32].

The qualitative part of the study was conducted in January 2024, following the completion of the first phase of quantitative data collection.

### 4.7. Data Analysis Method

Quantitative and qualitative data were analysed separately and compared in the integration phase.

#### 4.7.1. Quantitative Analysis

In the descriptive analysis, quantitative variables were described with mean and median as measures of centrality and standard deviation and interquartile range as measures of dispersion. Categorical variables were described with absolute values and percentages.

The Font‐Roja questionnaire was analysed by paired data. For the comparison of measures with paired (dependent) data, instead of working with the means of each group, we worked with their difference, since we were interested in knowing if this difference was zero or if, on the contrary, it was non‐zero. As the sample was *n* < 50, the Shapiro–Wilk test was used to verify the assumptions of normality, and the Student’s *t*‐test (normal distribution) or the Wilcoxon test (nonnormal distribution) was used. Values of *p* < 0.05 were considered statistically significant.

Missing values (missing or unknown) were coded when analysing the data. These were handled by deletion from the list, in accordance with standard practice in pre–post comparative analysis. The data (pre‐ and posttest) were collected in a database created specifically in Microsoft XP Access and analysed with IBM SPSS Statistics version 27.0 software.

#### 4.7.2. Qualitative Analysis

The data from the qualitative part were analysed through Braun and Clarke’s inductive analysis of the results [33]; therefore, the following six steps were followed: (1) familiarisation with the data; (2) generation of initial codes; (3) search for themes; (4) review of themes; (5) identification and naming of themes and (6) production of the report. The text was coded for meaning, and the codes were grouped into subthemes, which were then grouped into main themes. Two of the researchers carried out the coding, subcoding and development of the main themes. Finally, a third author reviewed the main themes; the authors discussed any discrepancies until a consensus was reached. The analysis was carried out with Atlas.TI.24 software.

#### 4.7.3. Mixed‐Method Analysis

The mixed methods analysis compared quantitative results with themes derived from the qualitative data. The results were analysed and interpreted to explore whether they extended our understanding and whether they were similar, convergent or inconsistent. Qualitative and quantitative data were analysed separately and compared in the integration phase. The results were integrated using a joint structure visualisation approach. The themes from the qualitative analysis were used to explain and contextualise the quantitative results, allowing us to assess whether or not the findings coincided.

### 4.8. Ethical Considerations

Approvals were obtained from the Centre’s Management and the Ethics Committee for Research on Medicinal Products of the Hospital Clínic de Barcelona (Spain) (Registration: HCB/2022/0732).

### 4.9. Research Rigour

The qualitative part of the study considered key criteria for assessing quality, including credibility, consistency, transferability and conformity [34–36]. Credibility was achieved through verbatim transcription of the interviews, ensuring that the terminology used by the participants remained unaltered. Consistency, another important criterion of reliability, was established through systematic procedures in data analysis, such as coding and the identification of common themes. In addition, techniques were used to verify the reliability of the analysis, such as intercoder or interrater consistency. Finally, transferability refers to external validity and the ability to apply the results beyond the context and sample of the study so that the findings are relevant to other settings [35–37].

## 5. Results

### 5.1. Quantitative Results

Finally, 36 subjects were included, representing 70.58% of the total of 51 ED workers, with 66.8% (*n* = 24) being female. The mean age was 39.17 (SD = 10.51) years. The median age was 38 years. Of these, 36.1% (*n* = 13) were nurses, 25% (*n* = 9) were physicians and 25% (*n* = 9) were auxiliary healthcare workers. The mean length of professional experience and experience in the ED was 13.64 (SD = 9.51) and 8.19 (SD = 6.07) years, respectively. A total of 63.9% (*n* = 23) were permanent staff. There was heterogeneity in participation according to work shifts (Table 2).

**TABLE 2:** Quantitative participants characteristics.

Characteristics	All participants (*n* = 36)
Age (year)	
Mean	39.17 (SD = 10.51)
Minimum	22
Maximum	58
Gender, *n* (%)	36 (100)
Female	24 (66.8)
Male	12 (33.3)
Length of professional experience (years)	
Mean	13.64 (SD = 9.51)
Years in the ED	
Mean	8.19 (SD = 6.07)
Job position, *n* (%)	36 (100)
Administrative	3 (8.3)
Auxiliary healthcare	9 (25)
Nurses	13 (36.1)
Physicians	9 (25)
Radiology technician	2 (5.6)
Work shift, *n* (%)	36 (100)
Mornings	5 (13.9)
Afternoons	9 (25)
Nights	7 (19.4)
Weekends	7 (19.4)
Rotating	8 (22.2)
Type of employment contract, *n* (%)	36 (100)
Permanent	23 (63.9)
Nonpermanent	13 (36.1)

Abbreviation: SD, standard deviation.

In terms of staff job satisfaction, as assessed by the Font‐Roja questionnaire, there was a statistically significant but modest increase in overall job satisfaction after LT implementation, 3.43 ± 0.41, compared to the preintervention overall job satisfaction of 3.31 ± 0.44 (*p* = 0.029). Not all individual dimensions showed meaningful improvements, although there was a significant pre–post increase in job monotony (3.30 ± 0.74 vs 3.64 ± 0.82, *p* = 0.038) and physical work setting (3.31 ± 1.06 vs 3.75 ± 0.84, *p* = 0.007) (Table 3). Effect sizes were small for most dimensions (*d* = 0.01–0.16), indicating minimal practical impact, with moderate effects for job monotony (*d* = 0.44, 95% CI [0.09, 0.78]) and physical work environment (*d* = 0.46, 95% CI [0.12, 0.80]). Overall job satisfaction increased with a small‐to‐moderate effect (*d* = 0.29), although the wide confidence interval (95% CI [−0.07, 0.64]) indicates considerable uncertainty in the estimate.

**TABLE 3:** Font‐Roja questionnaire results.

Dimensions	*N* pre/post	Preintervention Mean (SD)	Postintervention Mean (SD)	Test	Statistic	*p* value	Cohen’s d	95% CI
D1: Job satisfaction	36/36	3.86 (0.60)	3.93 (0.73)	Student′s *t*‐test	−0.775	0.443	0.10	[−0.22, 0.43]
D2: Work‐related stress	33/36	2.61 (0.55)	2.69 (0.65)	Student′s *t*‐test	−1.474	0.150	0.13	[−0.21, 0.48]
D3: Professional competence	35/36	3.67 (0.70)	3.73 (0.67)	Student′s *t*‐test	−0.249	0.805	0.09	[−0.24, 0.42]
D4: Job pressure	36/36	3.29 (0.96)	3.44 (0.93)	Student′s *t*‐test	−1.231	0.227	0.16	[−0.17, 0.49]
D5: Professional promotion	36/36	2.99 (0.81)	3.00 (0.73)	Student′s *t*‐test	−0.091	0.928	0.01	[−0.31, 0.34]
D6: Interpersonal relationship with superiors	36/36	3.79 (0.86)	3.81 (0.87)	Student′s *t*‐test	−0.285	0.777	0.02	[−0.30, 0.35]
D7: Interpersonal relationship with co‐workers	36/36	4.22 (0.92)	4.33 (0.71)	Wilcoxon^†^	103.5	0.406	—	—
D8: Extrinsic characteristic of status	35/36	2.78 (0.91)	2.86 (0.81)	Student’s *t*‐test	−0.706	0.485	0.09	[−0.24, 0.42]
D9: Job monotony	36/35	3.30 (0.74)	3.64 (0.82)	Student’s *t*‐test	−2.163	0.038	0.44	[0.09, 0.78]
D10: Physical work environment	36/36	3.31 (1.06)	3.75 (0.84)	Student’s *t*‐test	−2.859	0.007	0.46	[0.12, 0.80]
Overall job satisfaction	31/31	3.31 (0.43)	3.43 (0.41)	Student’s *t*‐test	−2.298	0.029	0.29	[−0.07, 0.64]

*Note:* Student’s *t*‐test for paired samples; Cohen’s d calculated for paired samples as (Mean_post–Mean_pre)/SD_pooled. 95% confidence intervals (CI) calculated for Cohen’s d. Effect sizes not calculated for nonparametric tests (D7).

Abbreviation: SD, standard deviation.

^†^Wilcoxon signed‐rank test for paired samples.

In some dimensions, the number of completed responses differed between pre‐ and postmeasurements due to missing questionnaire items. Only complete paired data were included for each comparison.

### 5.2. Qualitative Results

A total of 10 workers were included, six nurses and four physicians. Except for one, all of them were women. The mean age was 38.80 (SD = 8.76) years. The median was 40.50 years. The mean length of work experience and ED experience was 13.20 (SD = 8.21) and 8.70 (SD = 5.59) years, respectively. A summary table of main themes, subthemes, and representative quotes is provided to facilitate readability (Table 4).

**TABLE 4:** Qualitative themes and subthemes.

Theme	Subtheme	Representative quote
Lean Thinking (LT)	First impressions of LT	“Initially I was very reticent about the training, in fact, I was not a supporter and I didn’t like it and I said so clearly”. T10
	LT as a more methodical way of working	“The previous system was that they were arriving. That is, as the patient was arriving, he or she would go into the cubicle and you would see him or her when you could. So sometimes you would see one patient, see the other and ask for both tests at the same time. And that was a bit chaotic, wasn’t it? And it was more likely that there would be mistakes between one and the other”. T1
	Adaptation and re‐evaluation of LT	“I don’t think it is a bad method, Lean method, but I think it has to be adapted to each situation and to each and every place where it is implemented”. T7
	Work dynamics and interpersonal relationships	“Now I think that with Lean at the beginning, not now, but at the beginning it was, yes, there was quite a lot of conflict of… of each one who works…, what their work is, when they have to do it, with my team and with the doctors, for example”. T7
	Patient care	“I think so. It is true that many times they are not used to… to waiting times for nothing, so to speak, because every 20 min a patient passes and that’s how it is and it happens every 20 min and it can’t happen every 10 min. But I mean, that’s only at the entrance to the assessment, once you are inside, things are much more dynamic and go much faster and it is also much better organised so you can give your information to relatives, give them information, you know exactly what point, what point they are at and I think that is much more beneficial for the patient”. T2
	Would not go back to the previous way of working	“No, not right now, because I think it works well, it has its limitations but like everything else I guess. But… but no, the truth is that I think it works well and in a way it helps everyone to do things when they need to be done. And well, no, the service doesn’t collapse so much. Although sometimes that’s unavoidable”. T5

Job satisfaction	Work environment	“Look, for me, my key things are the colleagues I work with. That we work as a team. They are more than colleagues. I feel very much at ease”. T3
	Providing quality care	“The most important thing for me is to be able to offer quality care to the patient”. T6
	Leadership	“It is very demoralising for me not to feel listened to and taken care of by my bosses. And yes, it affects me even… I would say that… it affects me at the performance level because… Also related to the previous question, what do I value the most? Well yes, I don’t know if it’s selfish or not, but I value the recognition of work very much”. T10
	Training	“I’ve been on a couple or 3 courses that… well… I really… liked them. I like having that option or being offered that option”. T3

Abbreviations: T = transcript; LT = Lean Thinking.

In relation to the findings on the experience and perception of care staff, two main themes emerged, LT and job satisfaction, each containing different categories and concepts.

#### 5.2.1. Theme 1: LT

The experiences in LT focused on six subthemes: (1) first impressions of LT; (2) LT as a more methodical way of working; (3) adaptation and re‐evaluation of LT; (4) work dynamics and interpersonal relationships; (5) patient care and (6) would not go back to the previous way of working.

##### 5.2.1.1. Subtheme 1: First Impressions of LT

At the beginning of the implementation of LT, the participants were unanimous in their rejection. Some experienced it as an imposition from the hospital management and did not like the idea of changing the way of working in the ED. During the interviews, they mentioned that their first impressions of LT were negative because they had been informed during the training workshop that LT originated from the automotive industry. This explanation did not go down well with the staff, who felt that patients could not be treated as if they were in a car factory.

“Well, I received it as an… imposed way of working, that we had to work this way. They didn’t ask our opinion or adapt the Lean way of working to the way we worked here. And… well, it was a model that they followed in a car factory and that we had to work in the same way”. Transcript 7

“Initially I was very reticent about the training, in fact, I was not a supporter and I didn′t like it and I said so clearly”. Transcript 10

##### 5.2.1.2. Subtheme 2: LT as a More Methodical Way of Working

When asked about the way they worked before the introduction of LT, the vast majority mentioned the lack of organisation in the ED. They commented that at times of high demand for care, patients would congregate in the corridors when the cubicles were full. This increased the burden of care on staff, who had to deal with a very large number of patients at the same time. This could lead to medical errors, stress and work overload. All participants commented that after the implementation of the LT, they started to work in a more orderly way, resulting in fewer errors and less stress. They felt that there was less ’chaos’. In addition, by not seeing more than 6 patients at a time, patient flow improved, and the ED stay was not longer than necessary.

“The previous system was that they were arriving. That is, as the patient was arriving, he or she would go into the cubicle and you would see him or her when you could. So sometimes you would see one patient, see the other and ask for both tests at the same time. And that was a bit chaotic, wasn’t it? And it was more likely that there would be mistakes between one and the other”. Transcript 1

“I think that …. in terms of the orderly way of working, we have improved”. Transcript 7

##### 5.2.1.3. Subtheme 3: Adaptation and Re‐Evaluation of LT

Regarding the professionals’ opinions on LT, all participants mentioned that for LT to work, it has to be adapted to the service in which it is implemented. In the interviews, they expressed that patients attending EDs in different centres could not be treated in the same way due to differences in infrastructure, staff and patients. They all expressed an interest in regular meetings to review the operation and see what aspects could be improved, changes made and patient care optimised.

“It′s not being polished, it’s not being adapted. I don′t know if it’s because of time that they need more time or that it’s going to stay like this. I don′t know. No, I don’t know, but I would like to see it simply reviewed again”. Transcript 10

“I don’t think it is a bad method, the Lean method, but I think it has to be adapted to each situation and to each and every place where it is implemented”. Transcript 7”

##### 5.2.1.4. Subtheme 4: Work Dynamics and Interpersonal Relationships

During the interviews, participants were asked whether working dynamics and team spirit had been affected by the implementation of the LT. In this regard, participants mentioned both positive and negative aspects. On the positive side, the health workers noted that it was easier for them to help their colleagues because of the improved organisation and having a set time for each task. It was also mentioned that there was more effective communication between nurses and medical staff. On the negative side, they noted that LT caused friction among staff, especially at the beginning of the implementation.

“Now I think that with Lean at the beginning, not now, but at the beginning it was, yes, there was quite a lot of conflict of… of each one who works…, what their work is, when they have to do it, with my team and with the doctors, for example”. Transcript 7

##### 5.2.1.5. Subtheme 5: Patient Care

Professionals’ perceptions of the impact of the introduction of LT on patients varied. Some said that it had benefited patient care because everything was more organised and fewer mistakes were made. Others felt the opposite. They felt that this new way of working had increased waiting times for patients. In addition, most participants mentioned that each patient had different needs and that not all patients could be treated in the same way. They said that LT should be more flexible and take into account that each patient may have a different and varied situation.

“I think so. It is true that many times they are not used to… to waiting times for nothing, so to speak, because every 20 min a patient passes and that’s how it is and it happens every 20 min and it can’t happen every 10 min. But I mean, that’s only at the entrance to the assessment, once you are inside, things are much more dynamic and go much faster and it is also much better organised so you can give your information to relatives, give them information, you know exactly what point, what point they are at and I think that is much more beneficial for the patient”. Transcript 2

“There is a clash for me, because of course, a patient is a person and theoretically one should adapt the time to the person and their needs. In Lean method theoretically all [patients] have the same time, regardless of the need”. Transcript 10

##### 5.2.1.6. Subtheme 6: Would Not Go Back to the Previous Way of Working

Another question was: Would you go back to the previous way of working? Absolutely all participants said no. However, some emphasised that changes were needed to improve the way the ED worked and to adapt it to the patient and the service.

“No, not right now, because I think it works well, it has its limitations… but like everything else I guess. But… but no, the truth is that I think it works well and in a way it helps everyone to do things when they need to be done. And well, no, the service doesn’t collapse so much. Although sometimes that’s unavoidable.” Transcript 5

“No, maybe I wouldn’t go back, but I would modify Lean and adapt it to the situation here”. Transcript 7

#### 5.2.2. Theme 2: Job Satisfaction

Regarding the theme of job satisfaction, during the data analysis, four subthemes emerged that professionals considered to influence job satisfaction: (1) work environment; (2) providing quality care; (3) leadership and (4) training.

##### 5.2.2.1. Subtheme 1: Work Environment

When asked about the factors influencing their job satisfaction, all respondents agreed that an important factor was companionship, the team they worked with and a good working environment.

“Look, for me, my key things are the colleagues I work with. That we work as a team. They are more than colleagues. I feel very much at ease”. Transcript 3

##### 5.2.2.2. Subtheme 2: Providing Quality Care

ED staffs were very concerned about patient care. Many mentioned that providing quality care had a positive impact on patient satisfaction. They put the patient first, being able to care for them and respond to their needs.

“The most important thing for me is to be able to offer quality care to the patient”. Transcript 6

##### 5.2.2.3. Subtheme 3: Leadership

Participants explained that recognition of their work was something that influenced their job satisfaction. Feeling listened to by coordinators and managers were very important.

“It is very demoralising for me not to feel listened to and taken care of by my bosses. And yes, it affects me even… I would say that… it affects me at the performance level because… Also related to the previous question, what do I value the most? Well yes, I don’t know if it’s selfish or not, but I value the recognition of work very much”. Transcript 10

##### 5.2.2.4. Subtheme 4: Training

Participants mentioned that congresses and courses were essential to their work as health professionals. Medical and nursing professionals stressed the importance of updating and adding to their knowledge. They highlighted congresses and courses as a strong factor in their professional development. They commented that there should be more opportunities in this area, both financially and in terms of work. Financial and time constraints were the barriers that made it difficult for them not to attend the congresses or courses they wanted to attend.

“I’ve been on a couple or 3 courses that… well… I really… liked them. I like having that option or being offered that option”. Transcript 3

“I think it is very much related to the training you have. With less training you will not be able to grow as a professional. If you train and take extra courses, and you can apply these courses to your daily working life, you will be able to grow. But of course, if you don’t train because you don′t have the money, then it’s the same thing”. Transcript 5

**FIGURE 2:**
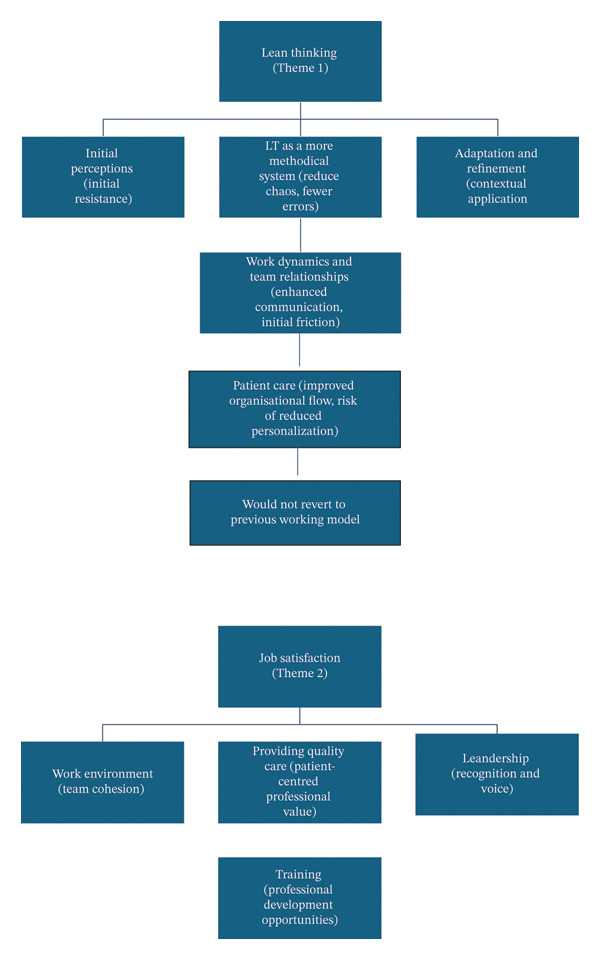
Thematic diagram.

The thematic diagram (Figure 2) visually illustrates the conceptual relationships between the two core themes and their subcomponents, demonstrating how staff perceptions of LT intersect with elements of job satisfaction.

### 5.3. Mixed‐Method Results

The results of the quantitative and qualitative parts complement each other. On the one hand, the quantitative phase found that job satisfaction had increased compared to the preintervention phase. The overall mean satisfaction before and after was compared using Student’s *t*‐test for related samples after normality; the difference was statistically significant. In the qualitative part, this increase in job satisfaction after the intervention also emerged. The in‐depth interviews in the qualitative phase allowed us to delve into the experiences of the professionals after the implementation of the LT, in particular their job satisfaction and the aspects related to it: the environment, knowledge, relationship with colleagues, working hours, organisation and lack of staff were the most frequently mentioned categories. While participants reported improvements in workflow clarity, professional engagements, and interpersonal collaboration, they also highlighted challenges including initial resistance to LT, concerns about potential dehumanisation of care and persistent staffing shortages.

During the personal interviews, professionals agreed on the difficulties they had in adapting to the new method, regardless of their professional role or employment contract. No differences were reflected based on subgroups according to role, shift pattern or contract type.

“Well (snort), yes, in the end it has improved because a more orderly way of working means that you have to take care of things that you didn’t before, that is, now you don’t take care of things that before you had to take care of or be aware of other things… In other words, you work more orderly and it is true that you work better”. Transcript 6

To support mixed‐methods integration, we constructed a joint display table linking key quantitative results with thematic qualitative findings (Table 5).

**TABLE 5:** Joint display table.

Finding	Quantitative evidence	Qualitative supporting quotation	Integrated interpretation	Implications for nursing management
Increase in overall job satisfaction	Overall satisfaction increased from 3.31 ⟶ 3.43, *p* = 0.029	“My level of satisfaction is much better… I am very comfortable in my job now.” (T2)	Staff report improved satisfaction, consistent with the measured increase	Reinforce conditions contributing to staff wellbeing and satisfaction
Reduction in job monotony	D9: 3.30 ⟶ 3.64 (*p* = 0.038)	“You work in a more orderly way and it is true that you work better.” (T6)	Structured workflow reduces monotony and boosts motivation	Continue refining role allocation and workflow clarity
Enhanced physical and organisational environment	D10: 3.31 ⟶ 3.75 (*p* = 0.007)	“We have improved in terms of working in a more orderly manner.” (T7)	More structured environment reduces stress and errors	Maintain Lean‐derived organisational improvements
Initial interpersonal tension	Not measured quantitatively	“At the beginning there was quite a lot of conflict…” (T7)	Change process caused transitional conflict	Support staff during implementation phases through communication and mediation
Initial resistance to LT	Not measured quantitatively	“I received it as an imposed way of working.” (T7)	Staff acceptance evolved over time	Engage staff actively in adaptation and implementation
Importance of teamwork	High baseline interpersonal relationship scores; slight improvement	“They are more than colleagues—I feel very much at ease.” (T3)	Team cohesion remains key to satisfaction	Promote interdisciplinary teamwork and supportive interactions
Centrality of providing quality patient care	Not directly measured in Font‐Roja	“The most important thing for me is to deliver quality care.” (T6)	Intrinsic motivation remains humanistic and patient centred	Balance Lean efficiency with personalised patient care
Need for continuous review and contextualisation of LT	Not measured quantitatively	“It has to be adapted to each situation and each place.” (T7)	LT must be flexible rather than rigid	Establish periodic revision of LT processes and feedback mechanisms

Abbreviations: LT, Lean Thinking; T, transcript.

## 6. Discussion

To the best of our knowledge, this study is one of the few mixed‐methods investigations assessing LT impacts on ED staff satisfaction in Europe. Some reviews have highlighted the paucity of mixed‐methods studies addressing this topic [9, 38]. On the other hand, there is a very limited number of studies on LT that specifically focus on the satisfaction and experience of healthcare professionals [19]. This underlines the importance of our study in providing a holistic view of the impact of LT, not only from an operational but also from a human perspective. The mixed‐methods design provides stronger evidence by explaining both the “what” (improved satisfaction scores) and the “why” (improved organisation, communication and need for adaptation).

Consistent with other authors, the results of this study indicated a significant increase in job satisfaction among healthcare workers 1 year after the implementation of the LT [39–41]. However, although satisfaction increased, the professionals emphasised during the interviews that they did not attribute this exclusively to the LT. This observation highlighted the complexity of job satisfaction and how it is influenced by multiple factors such as organisational support, working environment or training received.

For the most part, the implementation of LT has had positive effects on ED staff, but negative aspects have also been identified. In their interviews, professionals were very positive about the impact of LT on teamwork and communication among staff, although this impact was not significantly reflected in the quantitative part of the study, specifically in the dimension ’ interpersonal relationship with co‐workers ’ of the Font‐Roja questionnaire. LT improved team dynamics by establishing clearer and more efficient processes, as other authors have pointed out [39, 42]. On the contrary, some staff mentioned that the more structured organisation offered by the LT may limit the time available for individual patient care. This raises concerns about dehumanisation of care. Zibrowski et al. had already identified this perception of potential reduction in patient care quality among nurses [12]. These findings can be interpreted within broader theoretical frameworks of job satisfaction and change management. According to Herzberg’s Two‐Factor Theory, improvements in physical work environment and reductions in job monotony reflect enhancements in hygiene factors, which help reduce dissatisfaction, while motivational factors such as autonomy or recognition showed smaller changes [19]. The JD‐R model suggests that lean interventions can support staff engagement by providing additional resources to cope with workload, potentially explaining the moderate improvements observed in specific dimensions [43]. From a change management perspective, these results align with Lewin’s Change Model and Kotter’s 8‐Step Model, indicating that early‐stage Lean interventions may yield selective improvements. Broader effects on satisfaction, engagement, and resistance may require continued reinforcement, staff involvement, and organizational integration to achieve sustainable change [44].

In order for LT to be successful in the healthcare sector, it is important to recognise the significant differences between jobs in industry and jobs in the care sector [45, 46]. The commitment of the healthcare staff is essential for the successful implementation of LT in the ED. [11]. The success of LT depends not only on structural changes but also on the attitudes and commitment of healthcare workers. Our study showed that when staff feel valued and listened to and have the opportunity to actively participate in the improvement process, the implementation of LT can produce positive results in terms of both efficiency and job satisfaction. The professionals involved are willing and interested in holding regular meetings to make continuous improvements in the ED through the application of LT. Successful implementation is linked to active staff participation, supportive management and regular staff meetings [47]. Without adequate knowledge of lean management system and the necessary resources to support a continuous improvement process, positive results cannot be achieved. Sustained lean success depends on a change in the attitude and behaviour of leaders, which influences the unit’s staff and the organisational culture [48]. Increasing the commitment and knowledge of professionals can lead to less resistance to change, as reflected in our qualitative findings. Resistance to change in working methods on the part of staff is particularly evident when LT is imposed as a ‘non‐negotiable mandate, despite the dissatisfaction it may cause staff’ [49]. An authoritarian implementation of LT may be perceived by professionals as a rigid process that limits staff autonomy and compromises direct patient care due to lack of time [46].

Continuous improvement work in LT, coupled with the need to respond to complex and changing patient needs, can create a considerable workload for frontline workers and add significant responsibility to the management team [9]. Nevertheless, in our study, practitioners verbalised that the order offered by working with the LT gave them a sense of reduced workload. These findings underscore practical implications for nursing managements: adapting lean flexibly, scheduling regular follow‐up meetings, advocating for staff training and avoiding authoritarian implementation.

### 6.1. Strength and Limitations of the Work

There is no validated scale that addresses the satisfaction of healthcare workers regarding LT. For this reason, the Font‐Roja Job Satisfaction Questionnaire was used, which, although not specifically designed to assess LT, is considered a validated instrument with an internal consistency of 0.791 [29] for job satisfaction.

Another limitation of the study was the loss of the sample in the postintervention phase due to work‐related issues (sick leave or transfer of service) of the professionals. As the sample in the quantitative phase was smaller than expected, a second qualitative phase with in‐depth interviews was designed to complement the results of the first phase. Some of the reported benefits may also reflect a temporary novelty effect, as staff were newly exposed to lean practices. In addition, participants’ experiences were assessed 1 year after implementation, and the study lacks long‐term follow‐up, limiting insight into the sustainability of observed improvements. Furthermore, the study did not include a control group, which may limit the ability to establish causal relationships between the intervention and the observed outcomes.

Finally, as the study was conducted in a single, highly specific ED, findings may not be generalisable to other settings with different organisational characteristics or more mature lean cultures.

## 7. Conclusion

The level of job satisfaction of emergency professionals increased significantly, though modestly, after the implementation of the LT, primarily through improvements in work organisation, interprofessional communications, and opportunities for professional development. Staff expresses strong support for continuing lean while emphasising the need for ongoing adaptation, regular feedback cycles and mechanisms that ensure patient‐centred care. Although some participants expressed concerns about potential dehumanisation of patient care, structured processes were perceived as supporting safer and more reliable care.

The importance of expanding and continuing research in this area is highlighted in order to better understand the success factors and barriers to successful implementation, as well as the long‐term impact on patients, professionals and the institution. From a practical perspective, ED managers may benefit from involving frontline staff in the gradual refinement of lean interventions, offering accessible training opportunities to support familiarity with lean principles, and maintaining attention to patient‐centredness while streamlining processes. Future research should focus on multicentre and longitudinal studies to evaluate the sustainability over time, analyse the influence of leadership and organisational culture, and explore cross‐context or cross‐cultural comparisons to better understand key factors influencing lean implementation and its long‐term implications for staff and patient outcomes.

## Funding

The authors received no specific funding for this work.

## Conflicts of Interest

The authors declare no conflicts of interest.

## Supporting Information

Appendix 1: Value Stream Map of the Lean Intervention/Workshop Process.

## Supporting information


**Supporting Information** Additional supporting information can be found online in the Supporting Information section.

## Data Availability

Data available on request from the authors. The data that support the findings of this study are available from the corresponding author upon reasonable request.
