# Supplementary figures and images for: Improving Emergency Department Staff Satisfaction Through Lean Thinking: Evidence From a Mixed Study
Source: J Nurs Manag. 2026 Mar 20;2026:2158539. doi: 10.1155/jonm/2158539 (PMC13140332; doi:10.1155/jonm/2158539)

**Appendix 1: Value Stream Map**


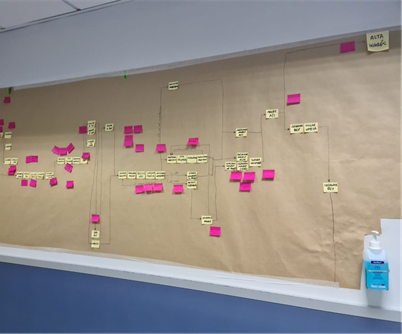

Supplement: Supplementary file 1 — Supporting Information Additional supporting information can be found online in the Supporting Information section. [file JONM-2026-2158539-s001.docx]
